# Investigation of avian influenza infection in wild birds in Ismailia and Damietta cities, Egypt

**DOI:** 10.14202/vetworld.2017.695-701

**Published:** 2017-06-25

**Authors:** Hanaa Mohamed Fadel, Rabab Afifi

**Affiliations:** 1 Department of Animal Hygiene and Zoonoses, Faculty of Veterinary Medicine, Suez Canal University, Ismailia, Egypt; 2 Department of Wildlife and Zoo Medicine, Faculty of Veterinary Medicine, Suez Canal University, Ismailia, Egypt

**Keywords:** avian influenza, hemagglutination inhibition, real-time reverse transcriptase-polymerase chain reaction, wild birds

## Abstract

**Aim:**

This study was carried out to monitor avian influenza (AI) infection in wild birds in Egypt.

**Materials and Methods:**

A total of 135 wild birds were examined for the presence of H5, H7, and H9 hemagglutination inhibition antibodies. Organs and swab samples of 75 birds were screened by multiplex real-time reverse transcriptase-polymerase chain reaction (RRT-PCR) to detect AI subtypes H5, H7, and H9 matrix genes.

**Results:**

The highest seropositive result was recorded in cattle egrets (90.9%) followed by crows (88.6%), semi-captive pigeons (44.8%), and moorhens (39.1%). In cattle egrets, semi-captive pigeons and moorhens, H5 antibodies predominated. In crows, H9 antibodies predominated. Multiple infections with two or three virus subtypes were highest in crows (6/39, 15.4%) followed by cattle egrets (3/30, 10%) and moorhens’ (1/9, 11.1%) positive samples. Multiplex RRT-PCR results revealed two positive samples in cattle egrets and moorhens.

**Conclusion:**

The results indicated high seropositive rates against AI virus subtypes H5 and H9 in the examined wild birds. Multiple infections with more than one AI virus (AIV) subtypes were detected in some birds. This requires a collaboration of efforts to monitor AIV infection in wild birds and implement suitable early intervention measures.

## Introduction

The spread of influenza viruses is a major cause of global concern for animal and public health [1]. Influenza viruses are members of *Orthomyxoviridae* family which consists of 3 genera, influenza A, B, and C viruses. Influenza Type A viruses are the only viruses reported to cause natural infection in birds. They are subtyped on the basis of characteristics of surface glycoproteins; the hemagglutinin (HA) and the neuraminidase (NA) proteins into 16 HA (H1-H16) and nine NA (N1-N9) subtypes [2]. According to their pathogenicity to poultry, they are divided into two groups, namely, highly pathogenic avian influenza (HPAI) and low pathogenic avian influenza viruses (LPAIVs). The HPAI viruses (HPAIVs) are restricted to strains that belong to H5 and H7 subtypes. They are lethal to chickens and turkeys and have a variable effect in water fowls and wild birds [3]. Most influenza A viruses originate from wild water fowls and shore birds, which are the primary reservoirs for these viruses [4]. Fouchier and Munster [5] reported that great antigenic and genetic similarities exist between H5 and H7 LPAIVs isolated from wild birds and those that caused HPAI outbreaks in domestic poultry in Europe. It was concluded that LPAIVs of the H5 and H7 subtypes represent HPAI precursors. The zoonotic transmission of AIV to humans occurs either directly from birds or from contaminated environments or through an intermediate host, such as pigs and wild birds [6,7]. There is no evidence suggesting sustained human to human transmission of the virus. However, H5N1 may mutate or reassort into a strain capable of efficient human-to-human transmission. Once occurs, a global emerging pandemic will threaten the human population everywhere.

The cumulative human case fatality rate for avian influenza A (AI) (H5N1) reported to the WHO from 2003 to 2015 from Canada and 15 Asian and African countries was 53.2%. In Egypt, 116 deaths were confirmed among 346 AI (H5N1) diagnosed cases representing a case fatality rate of 33.5% during the aforementioned period (WHO/GIP, data in HQ as of 13 November 2015). The Egyptian Ministry of Health and Housing reported WHO that the total number of AI confirmed cases from 2006 to December 2014 were 188 cases, of which 70 cases died. All cases had direct physical contact with infected birds. The sudden surge in the number of human infection with the H5N1 virus in Egypt, which began in November 2014 and continued through the winter months of 2015 is worrisome. During this period, the number of AIV cases has exceeded the amount of the country’s annual totals since the reemergence of human infection with the H5N1 virus in late 2003 (press data).

From December 2003, until now, HPAI H5N1 virus infection in birds has been reported in Middle Eastern, African, Asian, and European countries [8,9]. The infection with H5N1 has been reported in a diverse variety of wild avian species including quail, crow, and sparrow [10]. HPAI infection due to subtype H5N1 was first reported in poultry in Egypt in February 2006 [11]. In 2008, HPAI H5N1 virus became enzootic among poultry in Egypt. Moreover, Egypt has declared herself endemic for H5N1 to the OIE and continued notifying new cases in birds on a 6 monthly basis. In 2014, Egypt has reported the third highest number of poultry outbreaks globally [12]. Previous studies have documented the presence of other influenza A virus subtypes in migratory birds in Egypt, although none has reported isolating those viruses from domestic poultry [13]. Previous surveillance of AI from migratory birds from 2003 to 2009, in Egypt, revealed the isolation of H5N1 and H7N7 viruses from green-winged teal, northern shoveler, and northern pintail. Only one H5N1 virus was isolated in 2006 from a resident great egret [14]. In 2011, H9N2 virus was isolated from both quail and chicken farms in Egypt [15]. Investigation of wild bird infection might provide an early warning sign of potential novel AIV, circulating in the nearby poultry industry and even in human society because LPAIV infection of wild birds can evolve into HPAIV once introduced into poultry [16]. Thus, LPAIV circulating in wild birds pose an indirect threat to poultry and humans [17]. LPAIVs of the H9N2 subtype are particularly noteworthy due to their widespread circulation in domestic poultry ranging from the East to the Middle East [18]. Various influenza A (H5) subtypes, such as (H5N1), (H5N3), (H5N6), and (H5N8) have recently been detected in birds in Europe, North America, and Asia. Furthermore, AI virus (AIV) subtypes (H9N1) and (H9N2) were very common among domestic poultry in Egypt. Kayali *et al*. [19] documented the simultaneous cocirculation of H9N2 and H5N1 in poultry farms, human cases and the nearby environment in Egypt. The cocirculation of H9N2 virus with subtypes H5N1, H7N3, H1N1, and H3N2 can result in the emergence of a novel reassorted virus [20].

Therefore, this study was planned to detect AI infection in some wild birds in Ismailia and Damietta cities using hemagglutination inhibition (HI) and real-time reverse transcriptase-polymerase chain reaction (RRT-PCR) tests and to predict possible future outbreaks of AI infection in the study area.

## Materials and Methods

### Ethical approval

The study protocol was approved by the Local Department Council.

### Study area

The work was conducted during summer 2010 to winter 2013 in Egypt, namely, at Ismailia City which lies on the west bank of the Suez Canal, it is the capital of the Ismailia Governorate (Latitude: 30°36′15″ N and Longitude: 32°16′20″ E) and Damietta City which lies on the Mediterranean Sea and it is the capital of the Damietta Governorate (Latitude: 31°24′59″ N and Longitude: 31°48′47″ E), source: http://dateandtime.info/citycoordinates. A total of 135 wild birds were examined; house crow (*Corvus splendens -* 44), house teal (*Anas crecca -* 6), moorhen (*Gallinula chloropus* - 23), cattle egret (*Bubulcus ibis -* 33), and semi-captive pigeon (*Columba livia -* 29). The selection of species depended on the importance of these wild birds to the resident habitat. For example, the population of crows has dramatically increased in Suez Canal area which is associated with many ecological and epidemiological problems to the surrounding environment. Cattle egrets are considered “bridge” in the transmission of AIVs from poultry to wildlife and vice versa. As long as the aquatic wild birds are considered the primary reservoir of influenza A virus, the samples from moorhen and house teal were chosen.

The birds were handled in compliance with the American Veterinary Medical Association Guidelines on the Euthanasia of Animals [21].

### Capture

The moorhens were obtained from live wild bird markets in Damietta City. Pigeons (semi-captive) and house teals were obtained from Ismailia’s live bird markets (LBMs). Cattle egrets were hunted using traps, while crows were shot by a professional hunter from parks and areas near human habitation in Ismailia City.

### Serum

Blood samples were collected from 135 wild birds representing 5 birds spp. The blood samples (2-5 mL) were collected from wing vein using the appropriate sterile needles, syringes, and falcon tubes. After collection of the whole blood, it was allowed to clot by leaving it undisturbed at room temperature for 15-30 min. The clotted sample was refrigerated overnight. The clot was removed by centrifuging at 1000-2000 ×*g* for 5 min. The resulting supernatant (serum) was immediately transferred into clean, sterile polypropylene tubes using Pasteur pipettes and stored at −20°C until used.

### HI test

HI is considered the golden standard for AI diagnosis. HI test was applied to all available sera (135) to detect H5, H7, and H9 HI antibodies. HI titers (log_2_) were determined according to the standard method using chicken erythrocytes (0.5%) and four hemagglutinating units of virus (4 HAU/25 µl) [22].

### RNA extraction

A total of 75 birds’ samples were selected. The selection was based on HI test result. The samples comprised 25 individual birds (8 crows, 2 house teals, 4 moorhens, 5 pigeons, and 6 cattle egrets) and 25 pooled samples representing 50 birds (16 crows, 2 house teals, 9 moorhens, 11 pigeons, and 12 cattle egrets). Every two birds’ samples of the same species were pooled into one. RNA was extracted from organs, throat, and cloacal swabs of individual birds and pooled organs (trachea, lung, and intestine) using QIAamp viral RNA mini Kit (Qiagen, GmbH - Germany). The extraction was done according to the manufacturer’s instructions. Briefly, 140 µl of the sample suspension was incubated with 5.6 µl of carrier RNA and 560 µl of AVL lysis buffer at room temperature for 10 min. After incubation, 560 µl of absolute ethanol was added to the lysate. The sample was then washed and centrifuged following the manufacturer’s recommendations. Nucleic acid was eluted with 60 µl of AE elution buffer provided in the kit.

### RRT-PCR

Fifty samples representing 75 birds were selected for examination using multiplex RRT-PCR for detection of type A AI H5, H7, and H9 viruses targeting the matrix genes using primers and probes that were described by Slomka *et al*. [23,24] and Ben Shabat *et al*. [25]. Primer/probe sequences used for H5 were: 5’-3’ (H5LH1) ACA TAT GAC TAC CCA CAR TAT TCA G, (H5RH1) AGA CCA GCT AYC ATG ATT GC and (H5PRO) FAM-TCW ACA GTG GCG AGT TCC CTA GCA-TAMRA. For diagnosis of H7, the sequences were 5’-3’ (LH6H7) GGC CAG TAT TAG AAA CAA CAC CTA TGA, (RH4H7) GCC CCG AAG CTA AAC CAA AGT AT and (H7PRO11) HEX-CCG CTG CTT AGT TTG ACT GGG TCA ATC T-TAMRA. Primers/probes used for H9 diagnosis were 5’-3’ (H9F) GGA AGA ATT AAT TAT TAT TGG TCG GTA C, (H9R) GCC ACC TTT TTC AGT CTG ACA TT, and (H9 Probe) CY5-AAC CAG GCC AGA CAT TGC GAG TAA GAT CC -BHQ. DNA amplification was performed in a final volume of 25 µl containing 7 µl of RNA template, 12.5 µl of ×2 QuantiTect Probe RT-PCR Master Mix, 3.625 µl PCR grade water, 0.25 µl of each primer (50 pmol concentration) and 0.125 µl of each probe (30 pmol concentration) and 0.25 µl of QuantiTect RT Mix. Reverse transcription was done at 50°C for 30 min, primary denaturation at 94°C for 15 min, followed by 40 cycles of denaturation at 94°C for 15 s, annealing at 54°C for 30 s, and extension at 72°C for 10 s. The reaction was done in Stratagene MX3005P real-time PCR machine. The data were analyzed through Stratagene MX3005P software.

### Analysis of the PCR products

The amplified segments were separated by electrophoresis on 1.5% agarose gel (Applichem, Germany, GmbH) using 1x Tris/borate/ethylenediaminetetraacetic acid buffer at room temperature at gradients of 5 v/cm. For gel analysis, 15 µl of the products were loaded in each gel slot. 100 bp ladder (Qiagen, GmbH - Germany) was used to determine the fragment sizes. The gel was photographed by a gel documentation system (Alpha Innotech, Biometra).

HI test and RRT-PCR tests were done at the Reference Laboratory for Veterinary Quality Control on Poultry Production, Animal Health Research Institute (Central Laboratory and Ismailia Branch), Egypt.

### Statistical analysis

Geometric mean titers (GMT) for H5, H7 and H9 HI antibodies was calculated by using Excel software. Comparison of HI titer means was done using Tukey’s Kramer multiple comparisons and ANOVA tests, using SPSS software version 20. The p value was set at 0.05.

## Results

The highest seropositive result was recorded in cattle egrets (Table-1), with the predominance of H5 HI antibodies; (29/33, 87.9%) of the examined samples. Multiple infections with H5 and H9 HI antibodies represented (3/33, 9.1%) of the examined cattle egret samples (data were not shown in table). Regarding crows, H9 HI antibodies represented (38/44, 86.4%) of the examined crows’ samples. H7 antibodies were detected only in crows (3/44, 6.8%). Infection with H5 and H9 together represented (4/44, 9.1%) of the examined crows’ samples. H7 and H9 HI antibodies together were recorded in (1/44, 2.3%) of the examined crows’ samples (data were not shown in table). Moreover, multiple infections with H5, H7, and H9 together were detected only in a single crow sample. In semi-captive pigeons (44.8%), were positive; all were H5. In Moorhens (9/23, 39.1%), of the examined birds had H5 antibodies. Multiple infections with H5 and H9 represented (1/23, 4.3%) of moorhens’ samples (data were not shown in table). All the examined house teals were negative. The GMT for H9 HI antibodies was highest in crows (111.079) followed by cattle egrets (1.917) then moorhens (1.234). The GMT value for H7 HI antibody in crows was (1.17). For H5, the GMT values in cattle egrets were (93.4), pigeons (10.657), moorhens (6.676), and crows (1.506). The difference between means was extremely significant (F= 34.2, p<0. 0001).

**Table-1:** Evaluation of AIV infection in wild birds using HI test assay.

Bird species	Examined n	n (%)							

		Positive	H5^a^ only	H7^b^ only	H9^c^ only	Combined^a and b^	Combined^a and c^	Combined^b and c^	Combined^a,b and c^
House crow	44	39 (88.6)	-	1 (2.6)	32 (82.1)	-	4 (10.3)	1 (2.6)	1 (2.6)
House teal	6	-	-	-	-	-	-	-	-
Moorhen	23	9 (39.1)	8 (88.9)	-	-	-	1 (11.1)	-	-
Pigeon	29	13 (44.8)	13 (100)	-	-	-	-	-	-
Cattle egret	33	30 (90.9)	26 (86.7)	-	1 (3.3)	-	3 (10)	-	-
Total	135	91 (67.4)	47 (51.6)	1 (1.1)	33 (36.3)	-	8 (8.8)	1 (1.1)	1 (1.1)

a,b,c : refer to H5, H7 and H9 antibodies, respectively. AIV=Avian influenza virus, HI=Hemagglutination inhibition. The difference was extremely significant (F=34.2, p<0.0001).

**Figure-1:**
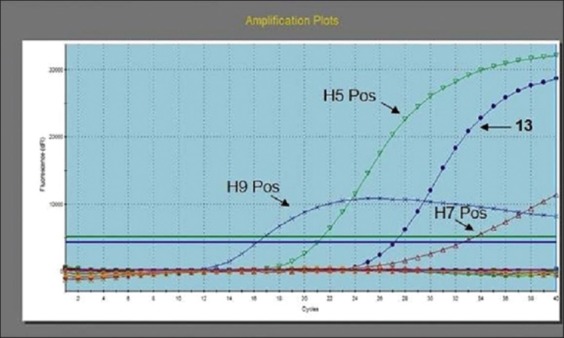
Avian influenza multiplex real-time reverse transcriptase-polymerase chain reaction amplification plot generated by Stratagene MX3005P software. The figure showed amplification of positive controls (H5, H7, and H9) in the three fluorescence filters FAM, HEX, and CY5, negative samples and positive curve of sample (13) for H5 gene at Ct 27.99.

Among the (50) RRT-PCR examined samples, two were positive (Table-2); one from pooled moorhens’ swab samples (Figure-1), and one from pooled egrets’ organ samples.

**Table-2:** Multiplex real-time RT-PCR results of AI infection in wild birds in relation to HI test results.

Samples	Individual samples		Pooled samples		Total examined RRT-PCR
		
	HI test +ve	HI test −ve	HI test +ve	HI test −ve	Number of samples (number of birds)
Examined RRT-PCR	21 (21 birds)	4 (4 birds)	19 (38 birds)	6 (12 birds)	50 (75 birds)
Positive RRT-PCR N (%)	-	-	2	-	2 (4)

RRT-PCR=Real-time reverse transcriptase-polymerase chain reaction, HI=Hemagglutination inhibition, AI=Avian influenza

## Discussion

AI is becoming one of the serious public health threats worldwide. Some AIVs are zoonotic [6]. The most well-known example is the AI subtype H5N1 which occurred in 1997 in chicken farms and LBMs of Hong Kong and resulted in the first documented case of human influenza infection and death attributable directly to AI [26]. The unusual outbreak of H5N1 HPAI in wild migratory birds in China in 2005 and the subsequent vast dissemination of the virus have thrown light on the role of wild birds in spreading the H5N1 HPAI virus [27]. The present study revealed multiple infections with several avian flu subtypes in crows, cattle egrets and moorhens. This gave a hint to the role of resident wild birds as vectors for influenza virus in the natural environment and may give a chance for genetic material to be exchanged between species-specific viruses. Pigs are considered the original “intermediate host” for influenza viruses [6]. However, other hosts such as the wild birds appear capable of similar conifection [7]. They may support such reassortment. It is worth mentioning that the population of crow has dramatically increased in Suez Canal area. Crows adversely affect the natural biodiversity of regions, as well as human health, tourism, infrastructure, and general development [28,29]. In Egypt, backyard poultry industry as a small-scale poultry production is very popular and crow as an omnivorous bird eating what is available (e.g., insect, fish, eggs, nesting birds, and vegetables) finds a good accessibility for food from backyard poultry waste products, so the crow as a terrestrial bird plays an important role in disease transmission due to its natural residence around the human environment and its accession to the poultry habitats. In the present study, we detected H5, H7, and H9 HI antibodies in crows. Previous reports mentioned the isolation of (H5N1) from crows (*Corvus macrohynchos*) in India [30] and from two crow species; *Corvus splendus* and *C. macrohynchos* in Bangladesh [31]. These countries have almost the same environmental life style like Egypt, where high human population with dense backyard poultry production is prevalent. The high seropositive rate of H9 subtype may give an indication of the circulation of H9 virus in domestic poultry. Previous studies of the H9N2 virus revealed that it has undergone extensive reassortment with many AIV including HPAI H5N1 and H7N3 [32,33]. Infection with H9N2 had been previously reported in two studies that were conducted in Pakistan; in crow naturally by Khawaja *et al*. [34] and experimentally with Iqbal *et al*. [35]. This strongly supports the role of a terrestrial wild bird such as crows in transmission of the infection. Kayali *et al*. [19] detected the H9N2 virus from poultry in Egypt and the isolation was as a single virus causing infection or coinfection in the same bird with H5N1, in addition both H5N1 and H9N2 were detected at the same time in the environment in poultry fields and in human cases. In contrast, Hassan [18] isolated H5N1 and H9N2 viruses from chicken farms and H9N2 virus from backyard ducks. He could not detect influenza viruses in wild birds.

Others terrestrial birds species such as cattle egrets may act as a “bridge” in the transmission of AIVs from poultry to wildlife and vice versa [34,36].

The present study showed high seropositive rates against H5N1 in pigeons. This supports the conclusions of Klopfleisch *et al*. [37] that pigeons are not resistant to (HPAIV) H5N1 infection, and might be at least hypothetically involved in the transmission of AI. This was confirmed clearly with Mansour *et al*. [38] in Egypt when they isolated (HPAIV) subtype H5N1 from mortality outbreak of pigeons that showed nervous manifestations and greenish diarrhea. The same clade of H5 virus (clade 2.2.1/C) was concurrently circulating in backyard poultry flocks and ducks and human cases. In Mosul, Iraq Al-Attar *et al*. [39] found that 81.8% and 50% of the examined wild pigeons were positive for H9N2 AIV virus using ELISA and HI tests, respectively. They concluded that pigeons may play an important role in spreading (AIV) as natural carriers. On the contrary, Perkins and Swayne [40] pointed out that pigeons do not contract or spread the virus. In conclusion, pigeons seem quite resistant to infection with AIV normally. However, it may be possible for AIV to become adapted to pigeons that make them a potential AIV host.

Regarding the moorhen, it is an extremely versatile species that is capable of occupying a diversity of freshwater habitats. It may wander away from water onto dry grassland, agricultural land, thus allowing the spread of AIV to backyard birds. In this present study, we could detect H5 and H9 HI antibodies in moorhens. This contradicted with El-Zoghby *et al*. [41] in their surveillance for A/H5N1 virus during the period 2006-2007 in Egypt; they detected A/H5N1 from 0.1% of the examined commercial poultry farms, 10.5% of backyard birds and 11.4% of LBMs but no wild bird tested positive for A/H5N1. Among the examined wild birds, they tested 25 common moorhens. This gives an indication for how much the virus endemically progresses and collaborates with the role of resident wild bird for that. On the other hand, Mehrabanpour *et al*. [42] detected LPAIVs (H9 subtype) by RT-PCR and virus isolation from migratory and wild resident birds that were examined in Boushehr, Iran.

In the present study, the high seropositive rates of wild birds against AIV that were detected using HI test did not agree with the RRT-PCR results except in two samples. The contradiction between serologic and PCR results was also reported by El-Zoghby *et al*. [43]. They could not detect AIV in swabs that were examined by RRT-PCR. On the other hand, Saad *et al*. [44] found that 15.57% of the examined migratory birds in Egypt were positive for influenza A virus matrix gene when tested by real-time PCR.

## Conclusion

Our findings indicated high seropositive rates against AIV subtypes H5 and H9 in wild birds from 2010 to 2013 in Egypt. Moreover, multiple infections with more than one AIV subtypes were detected in some birds. These paramount the important role played by these birds in the dissemination of AIV and set off alarm bells for the possible reassortment of these viruses in wild birds. Overall, regular monitoring of wild birds should be adopted to predict and prevent possible AIV outbreaks.

## Authors’ Contributions

The research was equally funded by the two authors. HMF was responsible for collection of samples, statistical analysis, performance of most of the experiments, and writing of the manuscript. RA collected samples, performed HI test, and shared in writing the manuscript. The manuscript has been revised and approved by both authors.
